# Weakly supervised large-scale pancreatic cancer detection using multi-instance learning

**DOI:** 10.3389/fonc.2024.1362850

**Published:** 2024-08-29

**Authors:** Shyamapada Mandal, Keerthiveena Balraj, Hariprasad Kodamana, Chetan Arora, Julie M. Clark, David S. Kwon, Anurag S. Rathore

**Affiliations:** ^1^ Department of Chemical Engineering, Indian Institute of Technology Delhi, New Delhi, India; ^2^ Yardi School of Artificial Intelligence, Indian Institute of Technology Delhi, New Delhi, India; ^3^ Department of Computer Science and Engineering, Indian Institute of Technology Delhi, New Delhi, India; ^4^ Henry Ford Pancreatic Cancer Center, Henry Ford Health, Detroit, MI, United States; ^5^ Department of Surgery, Henry Ford Health, Detroit, MI, United States

**Keywords:** pancreatic cancer, multi-instance learning, image segmentation, feature extraction, medical image analysis

## Abstract

**Introduction:**

Early detection of pancreatic cancer continues to be a challenge due to the difficulty in accurately identifying specific signs or symptoms that might correlate with the onset of pancreatic cancer. Unlike breast or colon or prostate cancer where screening tests are often useful in identifying cancerous development, there are no tests to diagnose pancreatic cancers. As a result, most pancreatic cancers are diagnosed at an advanced stage, where treatment options, whether systemic therapy, radiation, or surgical interventions, offer limited efficacy.

**Methods:**

A two-stage weakly supervised deep learning-based model has been proposed to identify pancreatic tumors using computed tomography (CT) images from Henry Ford Health (HFH) and publicly available Memorial Sloan Kettering Cancer Center (MSKCC) data sets. In the first stage, the nnU-Net supervised segmentation model was used to crop an area in the location of the pancreas, which was trained on the MSKCC repository of 281 patient image sets with established pancreatic tumors. In the second stage, a multi-instance learning-based weakly supervised classification model was applied on the cropped pancreas region to segregate pancreatic tumors from normal appearing pancreas. The model was trained, tested, and validated on images obtained from an HFH repository with 463 cases and 2,882 controls.

**Results:**

The proposed deep learning model, the two-stage architecture, offers an accuracy of 0.907  ±  0.01, sensitivity of 0.905  ±  0.01, specificity of 0.908  ±  0.02, and AUC (ROC) 0.903  ±  0.01. The two-stage framework can automatically differentiate pancreatic tumor from non-tumor pancreas with improved accuracy on the HFH dataset.

**Discussion:**

The proposed two-stage deep learning architecture shows significantly enhanced performance for predicting the presence of a tumor in the pancreas using CT images compared with other reported studies in the literature.

## 1 Introduction

Pancreatic adenocarcinoma is currently one of the deadliest cancers, with an overall 5-year survival rate of approximately 11% (1). Signs and symptoms of pancreatic cancer are non-specific and thus have limited utility in early detection. Moreover, efficient screening tests for the early detection of pancreatic tumors do not currently exist. It is clear that the pancreatic cancer survival rate is likely to significantly improve if the cancer can be detected at an early stage where definitive treatment with surgery and systemic therapy can be offered (2). Computed tomography (CT) and magnetic resonance imaging (MRI) are two common screening modalities that can be better utilized for diagnosing pancreatic cancer. Recent advancements in artificial intelligence and radiographic imaging provide hope that there may be the opportunity to use the aforementioned modalities as early screening detection tests, especially for early pancreatic cancer detection (3–5). Automated medical image segmentation and classification have been extensively investigated in the image analysis community due to the fact that manual, dense labeling of large amounts of medical images is tedious and error-prone. Accurate and reliable solutions are desired to increase clinical workflow efficiency and support decision-making through fast and automatic extraction of quantitative measurements (6, 7).

In the field of biomedical image analysis, the analysis of pancreatic images has significant importance in clinical diagnosis and research, including a range of tasks: (1) segmentation of tumor region, (2) diagnosing the presence of cancer, and (3) clustering the region. This research primarily focuses on an integrated framework that has the potential to perform classification, segmentation, and clustering. Traditional fully supervised techniques require accurately annotated data, which is laborious, uncertain, and time-consuming. However, unsupervised methods extract features from unlabeled data and have limited application to high-level tasks such as image segmentation and classification. In this scenario, the proposed algorithm strikes a balance by leveraging the benefits of both supervised and unsupervised approaches. This paper addresses the effectiveness and efficiency of accomplishing high-level tasks with a minimum of manual annotation and to automatically extract fine-grained information from coarse-grained labels.

**Figure 1:**
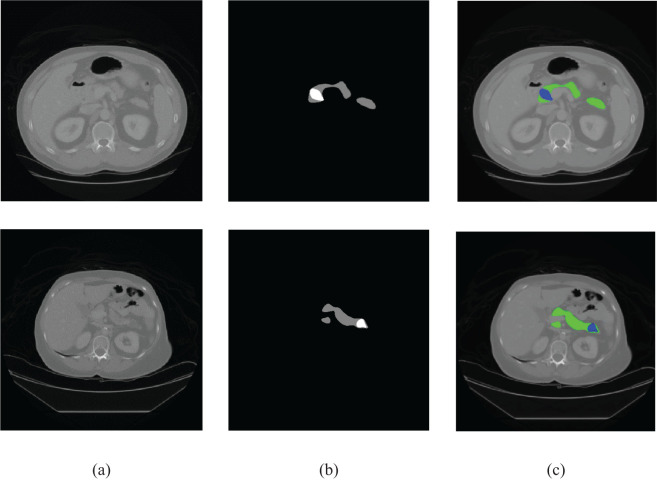
Sample CT images. **(A)** Original image, **(B)** ground truth, and **(C)** color-coded depiction of overlay on an axial CT image (pancreas: green, cancer: blue).

Convolutional neural networks (CNNs) (8) have made incredible strides in the medical imaging industry over the past 10 years, particularly in the areas of CT, MRI, and ultrasound image analysis. Since their advent, near-radiologist level performance has been achieved in automated medical image analysis tasks, including detection or prediction of hypertrophic cardiomyopathy (9, 10), future cardiovascular event (11), cancerous lung nodules (12, 13), liver tumors (14), and hepatocellular carcinoma (15). **Figure 1** demonstrates the difficulty in traditionally segmenting the pancreas compared with other organs due to various factors such as relatively small size, complicated anatomical structure due to adjacent structures, and uncertain boundaries of the organ in the limited slices in which the pancreas appears on traditional CTs.

High representation power and fast inference properties have made CNNs the *de-facto* standard for image segmentation and classification. Fully convolutional networks (FCNs) (12, 16) in general and U-Net (6) in particular are some of the commonly used architectures for automated medical image segmentation. The architectures are typically cascaded with multistage CNN models when the target organs show large inter-patient variation in terms of shape and size (17). Although deep learning has been studied for the detection of pancreatic cancer (18–24), pancreatic neuroendocrine tumors (pNETs) (25), and intraductal papillary mucinous neoplasms (IPMNs) in pancreas (26), it is yet to be incorporated as a part of the routine workup for patients diagnosed with pancreatic cancers. The U-Net model and its extended versions have been used in the literature for organ segmentation and have been demonstrated to deliver good accuracy (27–29).

**Figure 2:**
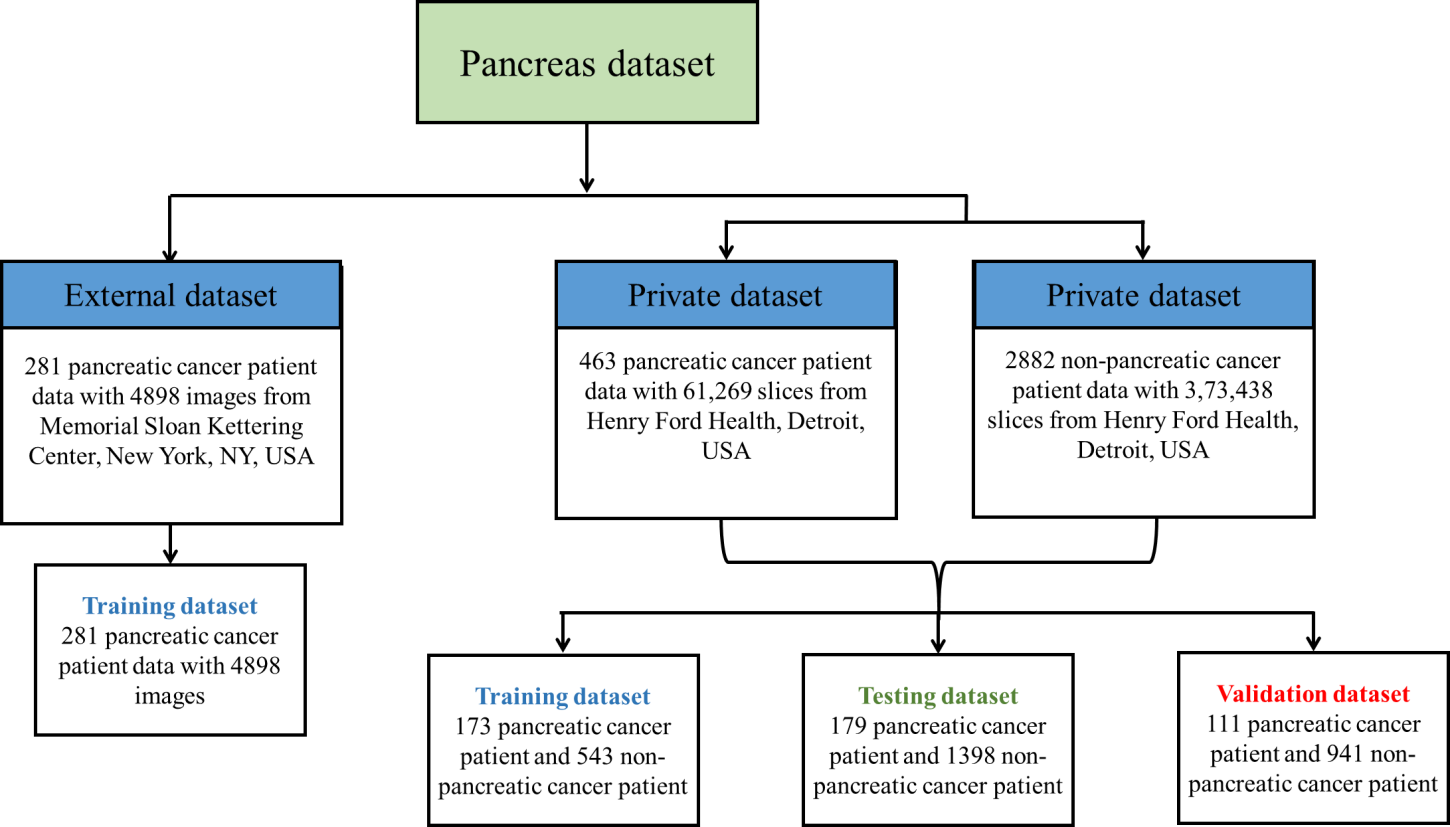
Private and external pancreas datasets used for model training.

Pancreatic cancer detection from CT images by applying deep learning is a challenging task because the pancreas is a small organ and is located in a complicated position in the retroperitoneum [Nakao et al., (30)]. A typical CT scan of a patient contains 131 slices in the full axial view, with approximately 20 to 60 slices containing the image of the pancreas. In early stages, the pancreatic tumors are too small and irregularly shaped for easy identification. Following that, several researchers proposed cutting-edge CNN techniques based on segmentation of the pancreas using either cascaded or coarse-to-fine segmentation networks. However, prior investigations of pancreatic segmentation were conducted on extremely limited populations (4, 18, 31, 32) and the results have been unsatisfactory (maximum dice coefficient 0.58) (33). This is primarily due to the minor differences between a singular image that contains the tumor vs. an image that does not. To the best of our knowledge, there are very few DL studies that have been conducted on big CT datasets that encompass a variety of pancreatic volumes. **Figure 2** illustrates the private and publicly available pancreatic dataset with normal and abnormal patients used for training the proposed framework. Therefore, the objective of this investigation is to carry out an effectiveness study using a weakly supervised algorithm with a total of 463 patients suffering from pancreatic tumors and a total of 2,882 controls.

Furthermore, as the tumor size is significantly small compared with the overall size of the CT image, the task of identification is further complicated when all slices are analyzed together. To circumvent these issues, we propose a weakly supervised two-stage architecture with a cascade of segmentation and classification. As we do not have annotated mask of the pancreas in our local dataset (Henry Ford Health (HFH)), the segmentation model was trained using MSKCC data to segment the pancreas. It should be noted that the Memorial Sloan Kettering Cancer Center (MSKCC) data set is small and does not capture all possible clinical variations of tumors. On the other hand, HFH is a much larger dataset but contains only patient-label information (cancer versus non-cancer). Our proposed approach intelligently combines “the best of both the worlds” by using a weakly supervised classification that utilizes patient label information of the HFH data and applies it to the cropped pancreas images, after initial processing by the segmentation model trained on the MSKCC data.

The goal of semantic segmentation (34) is to identify common features in an input image by learning and then to label each pixel in an image with a class. In this technique, raw image data are converted to quantitative, spatially structured information and can then be used for further processing. The segmentation method is an essential component in finding features in several clinical applications, such as applications of artificial intelligence in a diagnostic support system, tumor growth monitoring, therapy planning, and intraoperative assistance.

Based on FCN models, researchers have proposed a variety of strategies recently, including Hierarchical 3D FCN (35), DeepLab (36), SegNet (37), PSPNet (38), and RefineNet (39). The majority of these techniques automatically fit into the category of fully supervised learning methods, hence requiring a sufficient number of annotated data to train. Overall, the existing state-of-the-art methods for performing segmentation and classification either involve careful generation of handcrafted features or heavily rely on the extensive delineation of pancreatic tumor areas to give the annotation masks, which really place a tremendous load on the oncologists and researchers, respectively. Fully supervised methods have achieved remarkable performance in segmentation tasks such as brain tumor segmentation, lesion segmentation, and multiorgan segmentation (40). However, when a fully supervised algorithm is applied to pancreatic cancer detection and segmentation, these models have not achieved satisfactory results (3).

When employing advanced machine learning to the diagnosis of pancreatic tumors, the following major challenges arise: (1) Over 70% of pancreatic tumors have irregular shapes and ambiguous margins, resulting in imperceptible boundaries with the surrounding tissues. This characteristic increases the complexity of the segmentation process and may result in oversights when segmenting tumors. (2) The pancreas region is surrounded by many organs and tissues, and cancers affect a small area of the organ. Due to this, training a CNN architecture becomes difficult and the model gets distracted by irrelevant regions of the image, potentially leading to misclassification. (3) Training a deep learning model requires a substantial quantity of precisely annotated images for training. However, owing to the anatomical intricacy of the organ and differences in tumor appearance, physically identifying the pancreas and tumor locations is a labor-intensive and time-consuming task.

**Figure 3:**
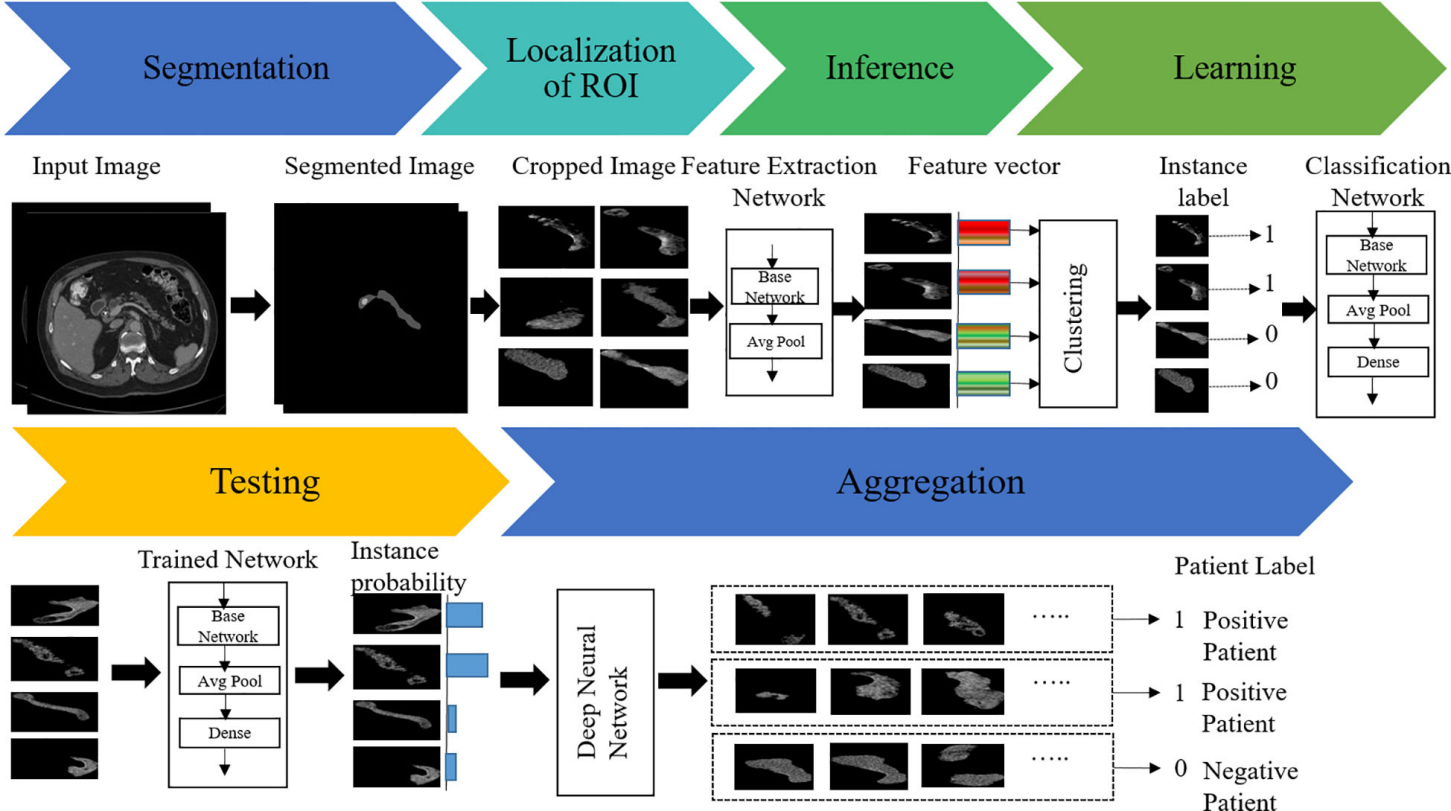
Illustration of the proposed two-stage architecture.

To overcome these issues, we herein propose a new weakly supervised algorithm that has a two-stage architecture, namely, segmentation and classification. In the first stage, the pancreas is segmented by the supervised segmentation model, and in the second stage, the multi-instance learning-based weakly supervised classification method is applied to the cropped pancreas images, which were obtained from segmentation, to classify pancreatic tumor images and normal-appearing pancreas, as schematically illustrated in **Figure 3**. There are three major impactful contributions from this work:

1. An end-to-end model for high-accurate pancreas segmentation along with classification has been proposed. The segmentation model is built upon an nn-Unet architecture, which segregates the pancreas, pancreatic cancers, and residual background organ and intraperitoneal space.
2. Furthermore, end-to-end multiple instance learning has been performed by multiple-instance neural networks, which accept a bag containing different numbers of instances as input and output the bag label right away.
3. Finally, comprehensive experiments on the unannotated HFH dataset have been conducted to demonstrate that the proposed approach outperforms other state-of-the-art techniques. To validate the effectiveness of the overall approach, the proposed architecture has been tested on a large volume of data obtained from the local data repository at Henry Ford Health (HFH) in Detroit, Michigan.

In this paper, Section 2 highlights the dataset used, network architectures, its components, and methodology. Sections 3 and 4 present the results and discussion in terms of ablation and comparison study. Finally, the conclusions of the study are presented in Section 5.

## 2 Materials and methods

CT images from HFH and Memorial Sloan Kettering Cancer Centre (MSKCC) were used to develop an end-to-end algorithm to detect the presence of pancreatic tumors in case versus control images. MSKCC is an open-source dataset that was used to train the segmentation model. Following this, we focused on the larger HFH dataset to further fine-tune and validate the model. A method has been proposed utilizing the axial view CT images and segmentation and classification techniques.

### 2.1 Dataset

In the proposed algorithm, the publicly available MSKCC dataset has been used to develop the segmentation model, followed by a separate and more robust HFH dataset used to train, test, and validate the MIL classification model. The MSKCC dataset comprised patients undergoing resection of pancreatic masses (31). These data consist of portal venous phase CT scans of 281 patients. Each patient has a single nifty (.nii) file that contains a full series of axial view images at the volumetric level. The classification model train, test, and validation data included CT images of 3,453 adult patients from HFH. Cases were images from patients diagnosed with pancreatic ductal adenocarcinoma, and controls were those where there was no suspicion of pancreas disease. Patients who had pancreatitis and women who were pregnant were excluded from the dataset.

### 2.2 Data preprocessing

In this study, retrospective imaging data were collected at Henry Ford Health (HFH), Detroit, Michigan, United States, from 2013 to 2020. Each patient on the HFH dataset had axial, sagittal, and coronal view images. In this investigation, the axial view is preferred because it provides a higher resolution and more detailed cross-sectional images of the pancreas. In addition, the axial perspective is consistent with the conventional clinical methods, which guarantees that our analysis and findings are reliable and consistent every time. For patients with known pancreatic tumors, image acquisition was performed with a pancreas protocol, high-resolution imaging cut at 2.5 mm with dedicated arterial and portal venous phases to identify vascular abnormalities, characteristics of hypoattenuating tumors, and to recognize hepatic metastases. The axial raw images were in DICOM format with several images (60 to 350) per study.

**Figure 4:**
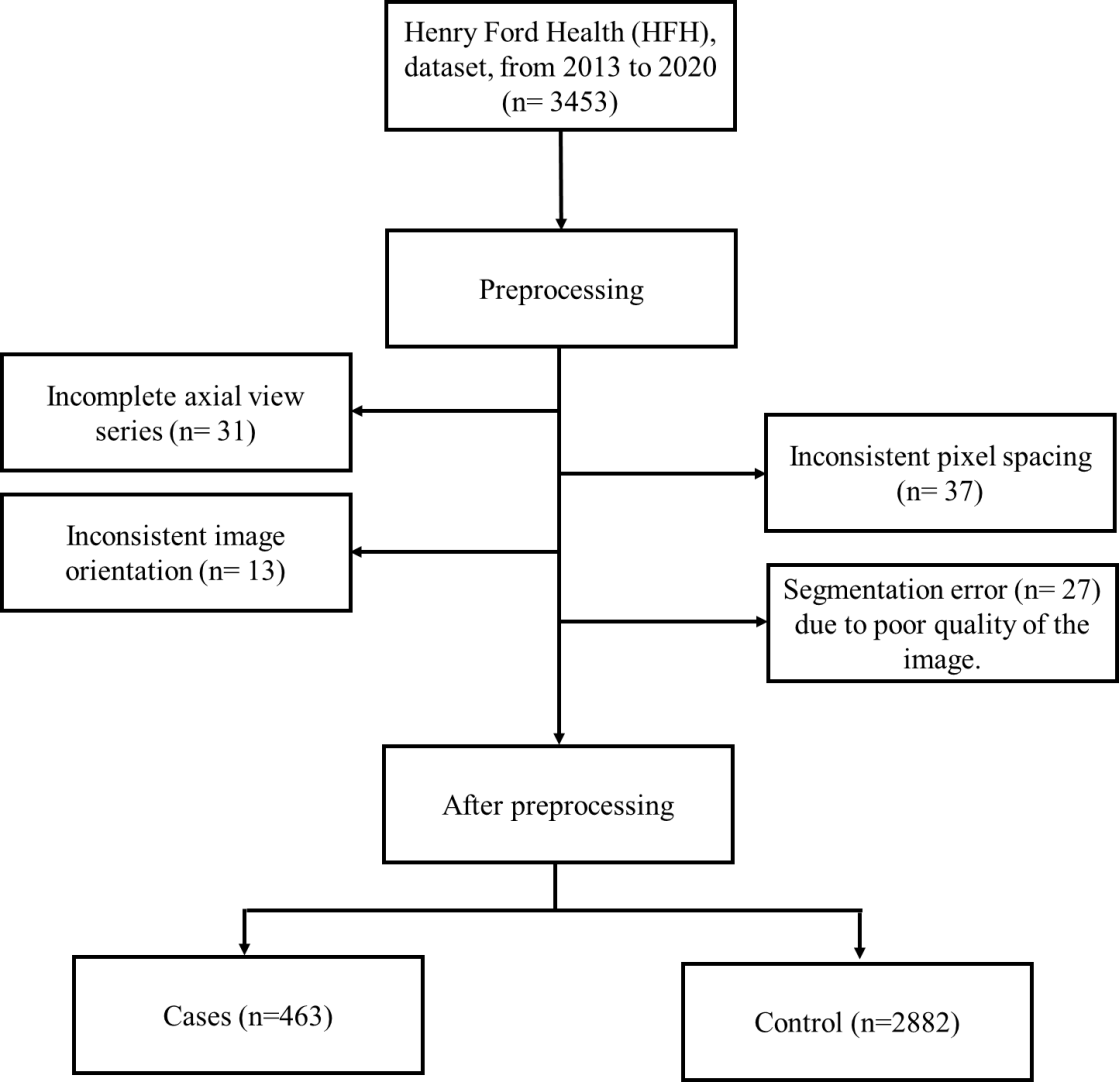
Patients’ enrollment and exclusion process in the HFH database.

A Python function was used to convert a 2D slice-level multiple images to a single 3D volumetric level image in nifty (.nii) format. To account for scanner and acquisition variability, a third-order spline interpolation was used for image data and nearest-neighbor interpolation for the corresponding segmentation mask to convert heterogeneous spacing to homogeneous (3, 41). During training and inference, each image was normalized using a global normalization scheme. While preprocessing HFH data, a total of 108 patients’ CT images were excluded due to incomplete axial view series (38), inconsistent pixel spacing (33), inconsistent image orientation (13), and segmentation error (29) due to poor quality of the image. This is represented in the flowchart (**Figure 4**). After this preprocessing step, the train, test and validation dataset sizes were 463 patients with pancreatic tumors and 2,882 controls.

### 2.3 Model architectures and training

In this study, the objectives of the segmentation method were to identify the slices containing the pancreas from the full-volume image and thereby identify the location of the pancreas in each slice. The segmentation method was applied to the volumetric axial view images, and it produced a segmented portion of the pancreas. In the first stage, the pancreas was segmented by the nnU-Net model (3). The segmented pancreas image was then used to find the location of the pancreas in the input image. The supervised classification model provides satisfactory results if the training data set captures a wide variation of the clinical samples. Therefore, we applied a weakly supervised classification model known as multi-instance learning (MIL). The MIL-based classification model was applied to distinguish pancreatic tumor and non-tumor pancreas on the cropped pancreas images. MIL is a weakly supervised classification wherein a label is only assigned to a collection of observations or a bag of instances (42–45).

In this approach, a three-dimensional image is converted into a bag of two-dimensional slices. Initially, each image is divided into patches as instance, with bags representing a subset of these patches. The instances are assigned with a label based on the presence or absence of the cancer region. If at least one instance within the bag consists of the tumor region, the bag is labeled as positive and none of the instances in the negative bag are positive. One of the challenges in applying MIL is finding the positive instances and negative instances from the positive bag. If all instances within the bad are classified as non-tumors, then the bag is labeled as negative. For the given coarse-grained (bag) label, MIL aims to predict the fine-grained (instance) labels for each patch within the image.

**Figure 5:**
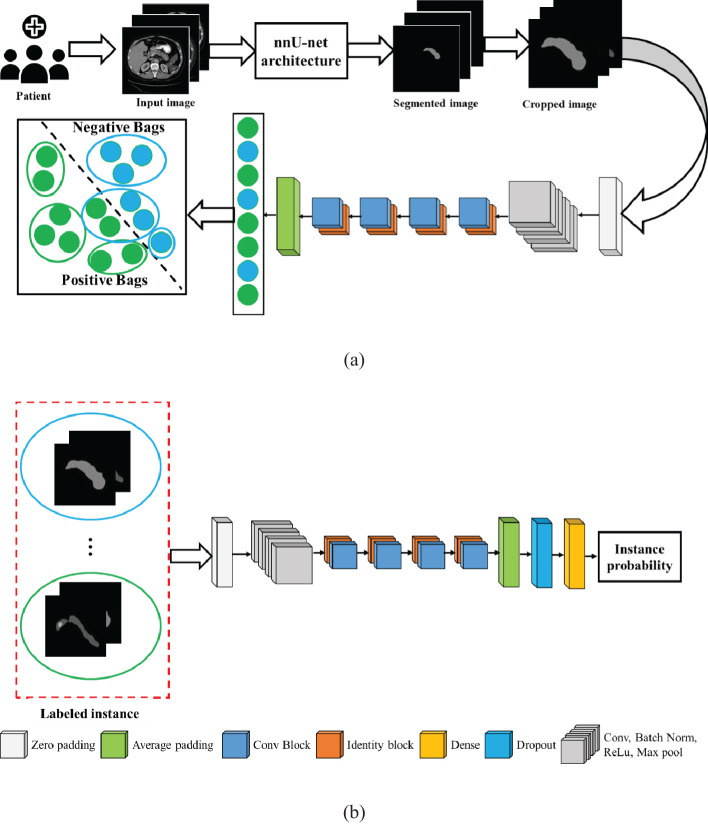
Architecture of the proposed model: **(A)** multi-instance learning, and **(B)** classification model.

To guarantee that each positive bag consists of more than one positive-labeled instance, the K-means clustering algorithm is employed to partition the data into distinct groups based on the features extracted from the images. The architecture of the proposed MIL approach is given in **Figure 5**. The feature of the instances was extracted by the average pool layer, where the base network is taken as ResNet50, a popular CNN framework used for image processing (46–48). This ensures that each positive bag consists of multiple instances representing tumors regions within the image. The following are the steps involved to obtain the predicted instance probability:

Step 1: We label the instances in a positive bag by clustering the image features into two groups: one is a positive group that contains all positive instances, and another is a negative group that contains negative instances. Given a dataset, ${\left\{X^{\left(i\right)},Y^{\left(i\right)}\right\}}_{i=1}^{N}$ containing N volumetric images, $X^{\left(i\right)}\in {\mathbb{R}}^{h_{i}\times w_{i}\times z_{i}}$ , and $Y^{\left(i\right)}\in \;\left\{0,1\right\}$ is the patient label, where 1 indicates positive patient and 0 indicates a normal patient. Each volumetric image $X^{\left(i\right)}$ is a bag of instances $\left\{x_{1}^{\left(i\right)},x_{2}^{\left(i\right)},\ldots ,x_{n}^{\left(i\right)}\right\}\in \;X^{\left(i\right)}$ .

Step 2: For the negative bag, all instances are negative. In the MIL pipeline, the features of the images are captured by ResNet50 and classified by ResNet50 (46).

Step 3: After extracting features of the images in ResNet50, K-means clustering was employed to get the label of the instance. In the feature extraction network, instance features are extracted at average pooling layers and returned as $\left\{\left(x_{1}^{\left(i\right)},f_{1}^{\left(i\right)}\right),\left(x_{2}^{\left(i\right)},f_{2}^{\left(i\right)}\right),\ldots ,\left(x_{n}^{\left(i\right)},f_{n}^{\left(i\right)}\right)\right\}$ , where $f_{1}^{\left(i\right)}$ is the feature of instance $x_{1}^{\left(i\right)}$ .

Step 4: The instance labels are assigned by clustering method as $\left\{\left(x_{1}^{\left(i\right)},y_{1}^{\left(i\right)}\right),\left(y_{2}^{\left(i\right)},f_{2}^{\left(i\right)}\right),\ldots ,\left(x_{n}^{\left(i\right)},y_{n}^{\left(i\right)}\right)\right\}$ , where $y_{j}\in \left\{0,1\right\}$ .

Step 5: The label instances are fed into the classification model that produces the predicted instance probability.

In this case, the ResNet50 model was used as a classification model. ResNet50 is a CNN-based classification method available in the Keras environment with pretrained weights in the TensorFlow backend. The original model was trained on the ImageNet dataset and was slightly modified in the proposed method. The fully connected output layers that were used for the prediction in the original model were not used. Instead, an average pooling layer with a pool size of (4 × 4) was added, followed by a dense layer with the number of neurons as 256. As the problem is that of binary classification, the output layer is a dense layer with dimension 2, and the softmax activation function is used for this layer. A dropout rate of 0.3 was applied in between the output layer and its previous layer. The final layer of the network, called the probability layer, calculates the probability of the input (cropped) image being of that class. Furthermore, a multilayer perceptron neural network (NN) was applied to aggregate the instance probability to patient probability and schematically illustrated in **Figure 5**. The NN structure is optimized, and the best-performing network is found to have one hidden with the number of neurons 18. The MIL and NN were trained by 173 cases and 543 control patients of the HFH dataset.

## 3 Results

To validate the effectiveness of the proposed architecture, a series of ablation studies with different baseline models were conducted in this section. The model was trained, tested, and validated on images obtained from an HFH repository with 463 cases and 2,882 controls.

### 3.1 Quantitative evaluation

We created a two-stage weakly supervised deep learning-based model to identify pancreatic tumors using CT images from publicly available Memorial Sloan Kettering Cancer Center (MSKCC) and Henry Ford Health (HFH) data sets. In the first stage, the nnU-Net supervised segmentation model was used to crop an area in the location of the pancreas, which was trained on the MSKCC repository of 281 patient image sets with established pancreatic tumors. In the second stage, a multi-instance learning-based weakly supervised classification model was applied on the cropped pancreas region to segregate pancreatic tumors from normal-appearing pancreas. The performance of the proposed two-stage architecture was then compared with the existing models that have been recently published.

**Figure 6:**
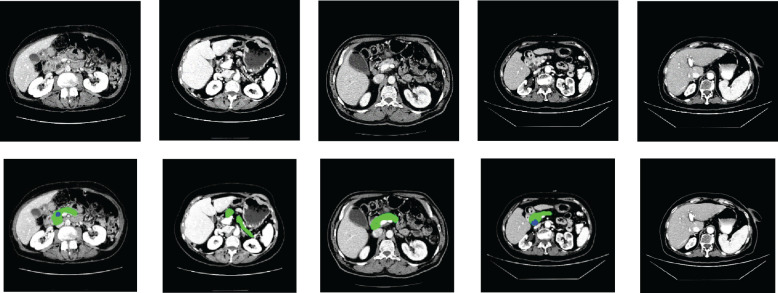
Segmentation results on HFH data: the first row represents the raw input image, and the second row represents the results of segmentation (pancreas: green, cancer: blue).

The performance of the proposed method was tested on the HFH dataset comprising 179 patients with known pancreatic tumors (23715 slices) as well as 1,398 patients without pancreatic cancer (182,757 slices), whereas the model parameters were fixed by the validation dataset including 111 patients with known pancreatic tumors (14,612 slices) as well as 941 patients without pancreatic cancer (122,214 slices), both selected randomly, yielding an average number of slices per patient of 131. A training dataset of HFH patients was created with 173 randomly selected patients with pancreatic cancer (22,942 slices) and 543 patients without pancreatic cancer (68,467 slices). The training dataset was used to train the MIL classification and NN aggregation models. The segmentation model was fed with the input CT image. The train and test data of HFH were segmented by the trained nnU-Net model. The nnU-Net model was trained by 281 patients of MSKCC dataset. **Figure 6** depicts the segmentation results of samples with both normal and abnormal by using the nnU-Net model applied on the HFH dataset. The **Supplementary Material** (**Supplementary Figure S1**) contains a box plot of the dice score for pancreas and pancreatic cancer regions.

The cropped pancreas images from the HFH train dataset were used to train the MIL classification model. In this case, only the list of positive patients and negative patients is known. In the MIL approach, each patient is considered as a bag, a cancerous patient is called a positive bag, and a control patient is called a negative bag. All instances in the negative bag are negative, and we directly fed the instances with label 0 to the classification model without clustering. In the positive bag, positive and negative instances are mixed. The instances in the positive bag are fed to the feature extraction model to extract the feature. The clustering method segregates the positive and negative instances by utilizing their features. The positive instances are labelled as 1 and fed to the classification model. The classification model was trained by the labeled instances from the training dataset. For test data, instance probability for the risk of cancer was predicted by the trained classification model. In the aggregation model, the neural network produces patients’ cancer probability by combining all instances of the probability of the patients. The neural network model parameters were fixed by the validation dataset. The results of validation dataset were as follows: sensitivity 0.847  ±  0.015, specificity 0.880  ±  0.025, 0.876  ±  0.021, and AUC 0.863  ±  0.01.

### 3.2 Ablation study of the proposed model

The proposed pancreatic tumor detection method was then compared with the existing nnU-Net-based segmentation method shown in **Table 1**. As the nnU-Net is a segmentation method for pancreas and pancreatic tumor segmentation (3), its efficacy toward identification of pancreatic cancer in the HFH dataset was characterized by measuring sensitivity, specificity, accuracy, and area under curve (AUC) based on the tumor segmentation results. The corresponding 95% confidence intervals (CI) were obtained using the Delong technique (49). With a 95% CI of (0.88, 0.92), the sensitivity was 0.905, demonstrating a high level of reliability in detecting true positives. The proposed framework’s ability to detect true negatives is reflected by specificity, which is 0.908 (95% CI: 0.86, 0.94). The model demonstrated outstanding performance across different assessment measures, with an overall accuracy of 0.907 (95% CI: 0.887, 0.927) and an AUC of 0.903 (95% CI: 0.883, 0.923).

**Table 1:** Comparison of the proposed model with different classifications on the HFH test dataset.

Model	Sensitivity	Specificity	Accuracy	AUC
nnU-Net	0.780 ± 0.03	0.801 ± 0.04	0.790 ± 0.03	0.791 ± 0.02
nnU-Net + MIL	0.831 ± 0.02	0.917 ± 0.01	0.908 ± 0.01	0.874 ± 0.02
**nnU-Net + MIL + NN** **(Proposed approach)**	0.905 ± 0.01	0.908 ± 0.02	0.907 ± 0.01	0.903 ± 0.01

The above mentioned results are tested on the HFH test dataset, which includes 179 cases (23,715 slices) and 1,398 control (182,757 slices). Data are sensitivity (95% CI).

**Figure 7:**
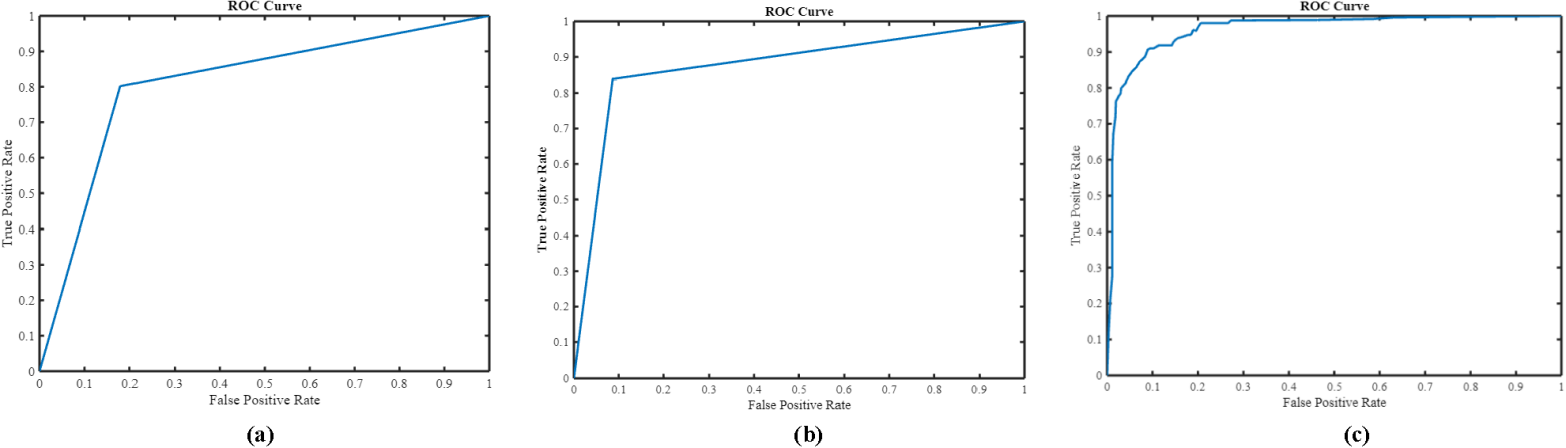
ROC curve on HFH test data: **(A)** nnU-Net model, **(B)** nnU-Net with MIL, **(C)** nnU-Net +MIL+NN.

As is evident, nnU-Net + MIL with the NN aggregation model (the proposed method) yielded the best performance with sensitivity 0.905  ±  0.01, specificity 0.908  ±  0.02 accuracy 0.907  ±  0.01, and AUC(ROC) 0.903  ±  0.01. In comparison, the nnU-Net + MIL and nnU-Net alone significantly underperformed, as shown in **Table 1**. The AUC(ROC) for the three methods are illustrated in **Figure 7**. As the nnU-Net + MIL + NN method provides each patient label as probability, the ROC is a smooth curve unlike other two methods which involve detection by setting threshold yielding binary patient label (0 or 1).

**Figure 8:**
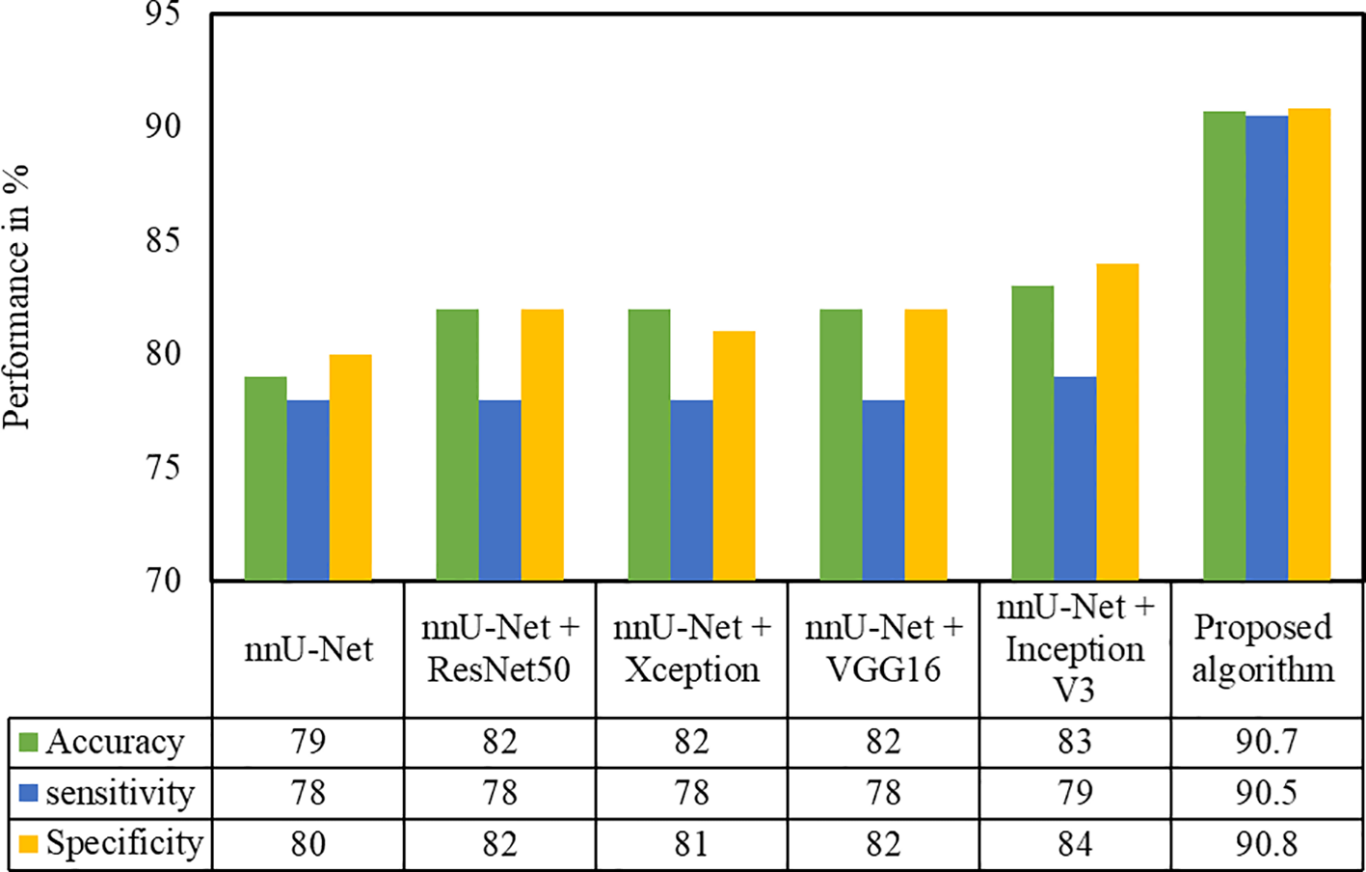
Performance evaluation of the proposed approach with different classification techniques on the HFH test dataset.

Several classification methods such as ResNet50, Xception, VGG16, and InceptionV3 were implemented in the second-phase test dataset on the cropped pancreas image. The pancreatic tumor detection results of these methods with the proposed architecture are depicted in **Figure 8**. It is evident that the proposed algorithm yields better performance in terms of accuracy and sensitivity. We have compared the proposed (nnU-Net + MIL with the NN aggregation model) architecture with various models such as nnU-Net. As can be deduced from **Figure 8**, the InceptionV3 classification model yielded the best performance (accuracy 0.83, sensitivity 0.79, and specificity 0.84) compared with the nnU-Net (0.79, sensitivity 0.78, specificity 0.8), nnU-Net + ResNet50 (accuracy 0.82, sensitivity 0.78, specificity 0.82), nnU-Net + Xception (accuracy 0.82, sensitivity 0.78, specificity, 0.81), and nnU-Net + VGG16 (accuracy 0.82, sensitivity 0.78, specificity, 0.82) models.

P-values are computed for both proposed and state-of-the-art techniques and are represented in **Supplementary Figure S2**.

## 4 Discussion

We have developed and validated an image analysis model that can identify pancreatic tumors on CT images by a combination of segmentation and classification methods, both based on deep learning techniques. The two-stage framework was developed utilizing a previously reported pancreas dataset from MSKCC and validated on the HFH dataset of control patients and pancreatic tumor patients. It has been demonstrated that the proposed method results in satisfactory performance. As is evident from **Table 1**, the proposed method yields superior performance when compared with other existing models and offers an accuracy of 0.907. Equally importantly, the performance of the proposed two-stage approach is demonstrated to be stable in a large dataset. The robust performance of the proposed method is an outcome of the verification and optimization that are performed at each layer. The information output from the pancreas is high in the segmented region. Hence, in the proposed method, the MIL classification method was applied only on the cropped pancreas image. As a result, the chances of identifying false positives are minimized. Overall, our results indicate that two-stage image analysis can distinguish between presence and absence of pancreatic tumors on CT images when given blinded images.

The nnU-Net used in this study has been applied toward medical segmentation decathlon (MSD) competition for multiple-organ segmentation and tumor detection tasks such as liver, spleen, kidney, pancreas, gallbladder, colon, and prostate (3). However, the detection efficacy of the pancreatic tumor by employing nnU-Net is limited, exemplified by a low dice coefficient of 0.53. In our study, we identified that a single-stage approach using nnU-Net produces more false positives, primarily because the contour of the pancreatic tumor is irregular and there are ill-defined margins on the CT image, leading to false detection of the normal pancreas portion as cancer. The CNN patch-based classification method was also attempted in literature for pancreatic cancers detection (18) but suffered from the following lacunae. First, CNN was trained to classify pancreas patches and pancreatic cancer patches. The patches were generated by the sliding windows method in a region of interest determined by the presence of pancreas fed in as the input. While this may be feasible in training, for a test image, such masks are unlikely to be available. Secondly, in this patch-based analysis, a patch is labeled positive, even if only a single pixel is predicted as cancerous. Apart from the above method, whole slide image (WSI) classification has been studied for pancreatic cancer detection (50, 51). Since the pancreas is a small organ, the tumor size is also very small in the early stages and thus a classification model alone is unlikely to be sufficient for locating the features of the small tumor portion with respect to the whole slide. This study also had limitations. Manual labeling of pancreatic images are labor-intensive, so we used publicly available datasets and private datasets for training and validation; the testing dataset included only American participants from a single institution. In response to this limitation, we used the MIL technique to balance both supervised and unsupervised approaches and verified the generalizability of the model. The findings demonstrate the effectiveness of a proposed two-stage weakly supervised deep learning system for detecting pancreatic cancer. By employing the proposed prediction model to aid in the radiographic diagnosis of tumors, therapeutic intervention may be accelerated, leading to better clinical results for the patient.

**Table 2** summarizes the state-of-the-art techniques validated on different datasets such as FLARE, NIH, and MSD. As indicated by the references in the table, the codes for these studies are not available, making it impossible to reproduce their investigations. Therefore, a direct comparison of these approaches is not feasible. However, all these studies used pancreatic images either for classification or segmentation tasks. A modified CNN model was trained to classify patches as cancerous or non-cancerous (18). The model was trained on the local dataset and externally tested on 281 patients with pancreatic cancer, and 82 individuals with normal pancreas. Researchers have developed a generalized pancreatic cancer diagnosis, and the method consists of anatomically guided shape normalization, instance-level contrastive learning, and a balance-adjustment strategy (23) on two unseen datasets (a private test set with 316 and a publicly available test set with 281). The effectiveness of the adaptive-metric graph neural network and causal contrastive mechanism has been developed to enhance the discriminability of the target features and improve early diagnosis stability (24). The training dataset with cross-validation consists of 953 subjects including 554 pancreatic cancer and 399 non-tumor pancreas. Despite the superior performance reported in the majority of the studies, a limited dataset with just a few hundred samples was utilized. We evaluated our pipeline on a significantly larger dataset with 463 cases and 2,882 controls CT images, whereby > 90% accuracy and sensitivity values were achieved. The significant diversity in the HFH dataset ensured strong generalization capabilities, demonstrating its superior performance. Moreover, our deployment of the segmentation model enabled both accurate normal pancreas and pancreatic cancer detection. As a result, our approach may enable an early detection modality that affords comprehensive options for clinicians to assess earlier onset of pancreatic cancers as well as offer curative intention options for pancreatic cancers that would not otherwise be feasible.

**Table 2:** Summarization of the existing techniques for pancreatic cancer detection.

Author	Dataset	Segmentation	Classification	Algorithm	Performance measure
Zhang et al., 2021 (52)	FLARE 2021	✓	✗	Efficient context aware network	DSC: 75.3
					NSD: 60.5
Wang et al., 2020 (53)	FLARE	✓	✗	Enhancement of pancreatic cancer using local and global multi-scale feature fusion	DSC: 79.5
					Jaccard: 66.6
Zhang et al., 2021 (47)	NIH and MSD	✓	✗	Lightweight deep convolutional neural network	Mean DSC: 84.90
					Min DSC: 61.82
					Max DSC: 91.46
Yu et al., 2018 (8)	NIH	✓	✗	Recurrent saliency transformation network	Mean DSC: 84.50
					Min DSC: 62.81
					Max DSC: 91.02
Oktay et al., 2018 (7)	NIH	✓	✗	Attention u-net	DSC: 82.2
Chen et al., 2022 (54)	NIH and MSD	✓	✗	Attention mechanism-based feature propagation and fusion	Precision: 85.6
					Recall: 85.9
					IoU: 74.8
Kim et al., (55)	MSD	✓	✗	Scalable gradient-based optimization	DSC 1: 80.61
					DSC 2: 51. 75
					NSD 1: 95.83
					NSD 2: 73.09
Liu et al., 2020 (18)	Private, MSD and TCIA	✗	✓	CNN with modified VGG network	Accuracy: 87.4
					Specificity: 86.7
					Sensitivity: 91.5
Li et al., 2023 (24)	Private	✓	✓	Adaptive-metric graph neural network and causal contrastive mechanism	Accuracy: 88.9
					Sensitivity: 88.7
					Specificity: 89.1
					AUC: 94.9
Qu et al., 2023 (23)	Private and MSD	✓	✓	Multiple instance learning and anatomically guided shape normalization	Accuracy: 89
					Sensitivity: 88
					Specificity: 89
					AUC: 94
**Proposed algorithm**	**MSD and HFH**	✓	✓	**nn-Unet and multi-instance learning**	**DSC 1: 81.64**
					**DSC 2: 52.78**
					**Sensitivity: 90.5**
					**Specificity: 90.8**
					**Accuracy: 90.7**
					**AUC: 90.3**

MSD, medical segmentation decathlon; NIH, National Institutes of Health; HFH, Henry Ford Health; DSC, dice similarity coefficients; IoU, intersection over union; NSD, normalized surface distance; AUC, area under the ROC curve. The symbol ✓ indicates that the reported paper used the method or result, whereas the symbol ✗ signifies that the reported paper does not include method, or result.

## 5 Conclusion

In this paper, we propose an end-to-end model for accurate pancreatic tumor prediction. The model incorporates segmentation using the nnU-Net architecture and multi-instance classification using weakly supervised learning. The pancreatic tumor samples are processed by localizing the area of interest from the segmented image. A bag is then formed for each region, which is labeled based on the grade. Finally, the multi-instance learning model is trained for classification. The proposed MIL classification technique achieves an optimal performance by utilizing patient label information on the cropped image, not on the whole pixel patches. Our experimental findings demonstrate that the proposed framework outperforms nnU-Net with Inception V3 by a large margin (7.0%) using the HFH test dataset. From the results, it is evident that the two-stage deep learning architecture of patient radiographic imaging has the potential to be of great assistance in the pursuit of early pancreatic tumor detection. Furthermore, it has the potential to reduce the number of incorrect diagnoses of pancreatic cancer, which would ultimately result in much-required improvements in patient care. We will investigate the possibility of employing auto-encoding DNN rather than K-means in the future.

## Data Availability

The datasets presented in this article are not readily available because CT images from HFH and Memorial Sloan Kettering Cancer Centre (MSKCC) were used to develop a novel algorithm to detect the presence of pancreas tumors in case versus control images. This data cannot be shared without patient approval. MSKCC is an open-source dataset that was used to train the segmentation model. Requests to access the datasets should be directed to DK, System Director, Surgical Oncology, Department of Surgery, Henry Ford Health, Detroit, MI, USA, dkwon1@hfhs.org.
