# Supplementary material for: Weakly supervised large-scale pancreatic cancer detection using multi-instance learning
Source: Front Oncol. 2024 Aug 29;14:1362850. doi: 10.3389/fonc.2024.1362850 (PMC11390448; doi:10.3389/fonc.2024.1362850)
Supplement: Supplementary file 1 [file DataSheet1.docx]

Supplementary Material

1. **Evidence before this study**

Pancreas adenocarcinoma is a deadly disease with significantly poor prognosis. In fact, by 2030, pancreas is predicted to become the 2^nd^ deadliest cancer in the United States. At this time, 80% of patients newly diagnosed with pancreatic cancer are found to be too advanced for curative intent. Moreover, early detection efforts have largely been ineffective thus far, owing to a multitude of factors, including the lack of effective screening tests as well as vague clinical signs and symptoms that alert physicians to the development of cancer. When symptoms do manifest, the cancer has already progressed to an advanced stage, by which time clinical or surgical interventions offer limited efficacy.

Radiographic imaging such as magnetic resonance imaging (MRI) or computed tomography (CT) has been the gold standard in diagnosis of pancreas tumours. The diagnostic performance of CT imaging for pancreatic cancer is interpreter-dependent, and approximately 40% of tumours smaller than 2 cm evade detection. This motivated us to explore the possibility if utilization of a two-stage deep learning architecture of patient radiographic imaging could possibly result in a more accurate method of early cancer detection that would otherwise potentially be missed.

A Pubmed search from July 2020 for research articles using the term ‘pancreatic cancer deep learning’, ‘pancreatic cancer machine learning’, and ‘pancreatic cancer convolution neural network’ was performed. Below, the most relevant works based on this query was notated. Convolutional Neural Networks (CNNs) have been applied to a wide variety of computer vision tasks. More recent advances in semantic segmentation have enabled their application to medical image segmentation, and its classification methods have been reported for detection of pancreatic cancer, risk stratification of intraductal papillary mucinous neoplasms, and pancreatic neuroendocrine neoplasms. Several case studies have been reported to analyse CT images using classification and segmentation algorithms for the organization of patients into those with pancreatic cancer and healthy individuals. At this time, only one case study has reported testing results on an independent external dataset. In this method, relevant patches of an image (CT slice) to be classified were generated based on the pancreas mask as the input. However, pancreas masks are not routinely available in most clinical settings and therefore the published approach offers limited applicability in practice.

1. **Added value of this study**

We have developed a two-stage architecture, with stages corresponding to segmentation and classification respectively, to correctly identify the presence of pancreatic tumours from controls on CT-based imaging. The proposed weakly-supervised model is a cascade of segmentation and classification and has been tested on CT image data obtained from a large repository from HFH and exhibited good performance.

1. **Implications of all the evidence**

These results demonstrate the efficacy of a proposed two-stage weakly-supervised deep learning framework for detection of pancreatic cancer. The two-stage framework can automatically differentiate pancreatic tumours from non-pancreas tumours with improved accuracy (from 78% to 90%). Employing a prediction model to assist with radiographic detection of tumours will enable a quicker clinical intervention, thereby improving the clinical outcomes for the affected patient.

1. **Inference**

In this study, owing to the proposed two stage approach, the segmentation method selected the region of interest by cropping the pancreas region and the classification was applied only the region of interest (pancreas). Therefore, the number of false positives were significantly reduced, and higher specificity could be achieved, compared to the single stage nnU-Net model. Since the mask of the salient (pancreas) region is not available for a test (HFH) image, the segmentation method is first applied to generate the mask. The proposed MIL classification method only uses patient label information on the cropped pancreas, and not the in whole pixel patches. Hence, the proposed model balances sensitivity and accuracy to achieve an optimal performance.

Patients diagnosed with pancreatic cancer have an unfortunate prognosis, with a median survival rate of eight months, specifically in pancreas adenocarcinoma. Currently, approximately only 20% of patients are found early enough for curative treatment, leaving the rest with valiant, but unfortunately, non-curative, palliative options. Additionally, few pancreatic cancer patients experience sustained therapeutic responses mainly due to the lack of early tumour detection. Thus, early detection of this dreadful disease is likely to have a significant impact on improving the dismal outcomes associated with pancreas adenocarcinoma.

In the current health care environment, radiographic imaging such as computer tomography is being utilized more frequently to facilitate the diagnosis and management of patients with a myriad of abdominal symptoms. With over 70 million outpatient and emergency CT scans annually performed, there is a wealth of data that would suggest the utility of leveraging this methodology to facilitate the early detection of pancreas tumours [1]. Moreover, integrated health care systems, such as Henry Ford Health, provide medical care over a continuum of time, and incorporating such technology with radiographic data that is longitudinal in nature provides a meaningful way to incorporate deep learning methods for patient care. It is understood that human error or subjective interpretations will continue to factor in missed diagnoses or the early identification of tumours. However, the promising results of utilizing a two-stage deep learning architecture of patient radiographic imaging could greatly benefit in the pursuit of early pancreatic tumour detection and using the proposed two-stage method as a second reader could reduce misdiagnosis of pancreatic cancer, which in turn would lead to much needed improved patient outcomes.


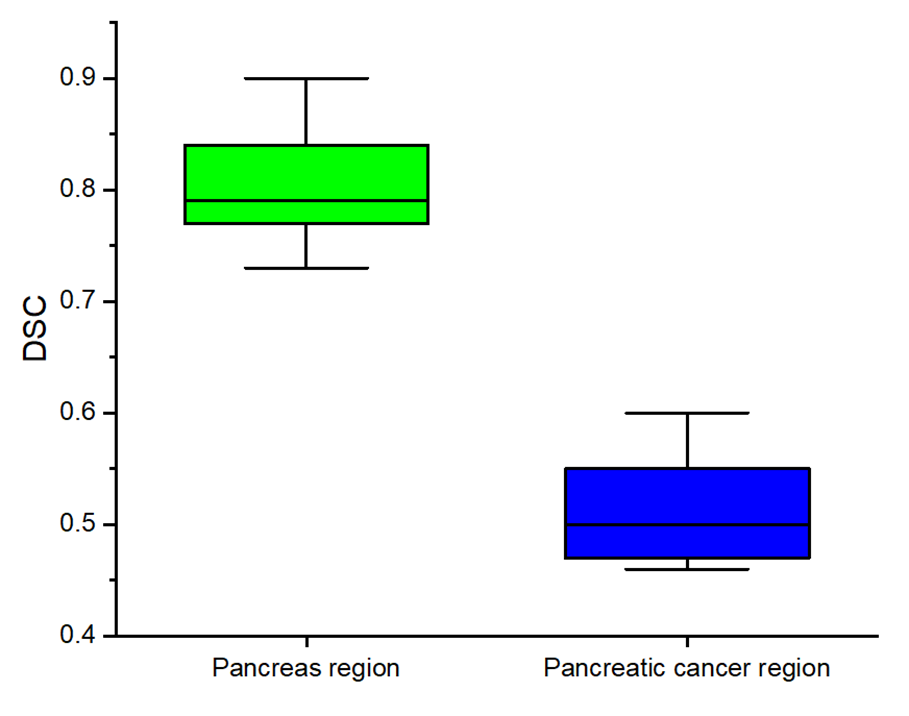


Figure S1. Box plot of test dataset with respect to DSC for pancreas and pancreatic cancer regions.

The dice similarity coefficient (DSC) is computed for both pancreas and pancreatic cancer region segmentation and the results are represented in a box plot (Figure S1). Using the Wilcoxon signed-rank test, we have carried out further statistical testing in order to determine the validity of the differences between the algorithms that were evaluated are statistically significant. P-values are computed for both proposed and state-of-the-art techniques and are represented in Figure S2.


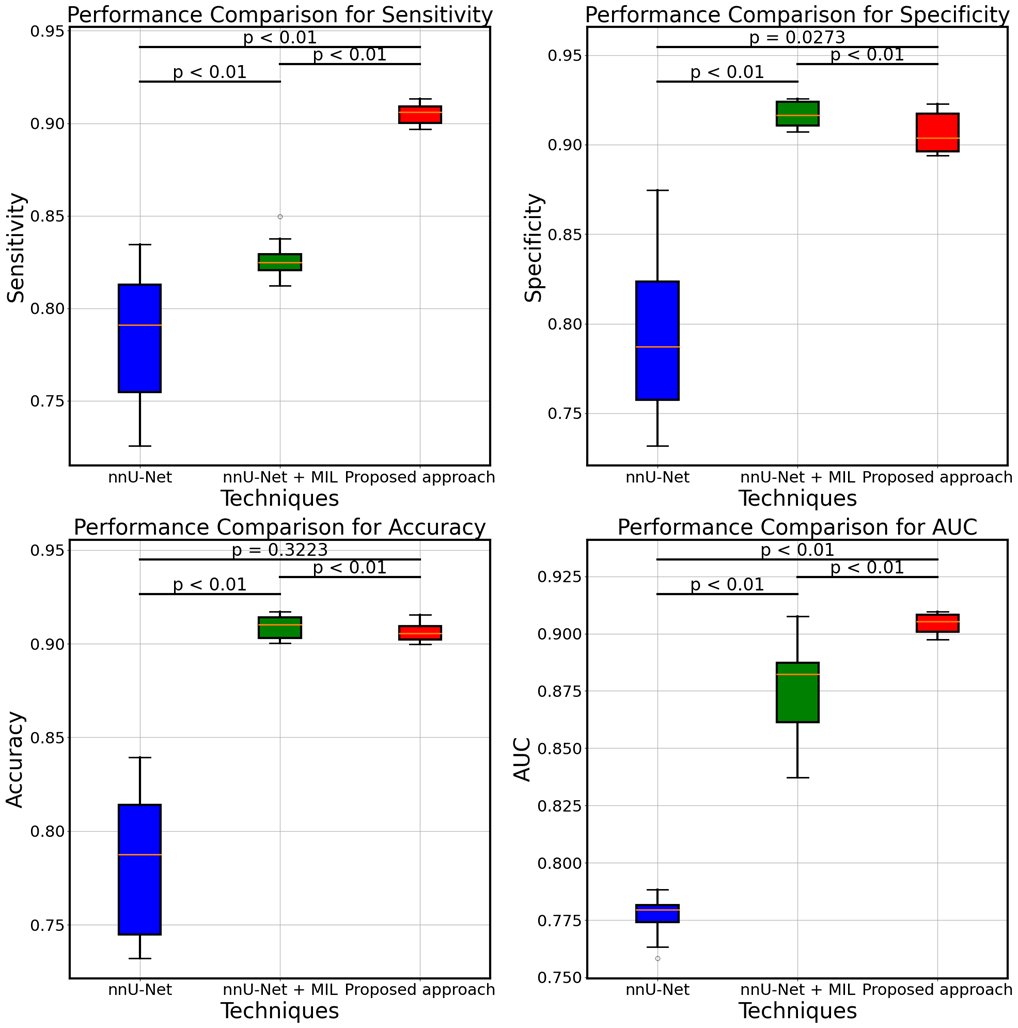


Figure S2. P-value of the proposed and state-of-the-art techniques.

Reference:

1. Bhatt A, Yang X, Karnik N, Sill A, Kowdley G. Use of computerized tomography in abdominal pain. Am Surgeon. (2018) 84:1091–6. doi: 10.1177/ 000313481808400671
